# Culture-Based Assessment of Presumptive Resistant Bacterial Taxa in the Urban Danube River near Novi Sad: Environmental Associations Revealed by Machine Learning

**DOI:** 10.3390/antibiotics15060553

**Published:** 2026-05-30

**Authors:** Miloš Jovićević, Dušan Kekić, Ana Tomić, Olja Šovljanski, Lato Pezo, Nemanja Mirković, Radmila Novaković, Ivan Vicic, Nikola Bajcetic, Milica Mirkovic, Nedjeljko Karabasil, Nataša Opavski, Ina Gajić

**Affiliations:** 1 Institute of Microbiology and Immunology, Faculty of Medicine, University of Belgrade, 11000 Belgrade, Serbia; milos.jovicevic@med.bg.ac.rs (M.J.); dusan_vk@yahoo.com (D.K.); natasaopavski@gmail.com (N.O.); ina.gajic@med.bg.ac.rs (I.G.); 2 Faculty of Technology Novi Sad, University of Novi Sad, 21000 Novi Sad, Serbia; oljasovljanski@uns.ac.rs; 3 Institute of General and Physical Chemistry, Studentski Trg 12–16, 11000 Belgrade, Serbia; latopezo@yahoo.co.uk; 4 Faculty of Agriculture, University of Belgrade, 11000 Belgrade, Serbia; nemanja.mirkovic@agrif.bg.ac.rs (N.M.); nikola.bajcetic@agrif.bg.ac (N.B.); petrusicm@agrif.bg.ac.rs (M.M.); 5 Institute of Molecular Genetics and Genetic Engineering, University of Belgrade, 11000 Belgrade, Serbia; radmila.novakovic@imgge.bg.ac.rs; 6 Department of Food Hygiene and Technology, Faculty of Veterinary Medicine, University of Belgrade, 11000 Belgrade, Serbia; ivan.vicic@vet.bg.ac.rs (I.V.); nedja@vet.bg.ac.rs (N.K.)

**Keywords:** antimicrobial resistance, Danube River, wastewater, environmental factors, machine learning, multidrug-resistant bacteria, water quality, One Health, XGBoost, Random Forest

## Abstract

**Background/Objectives**: Environmental dissemination of antimicrobial resistance (AMR) is increasingly driven by wastewater-impacted aquatic systems, yet the key factors controlling multidrug-resistant (MDR) bacterial distribution remain unclear. This study evaluated environmental factors associated with MDR bacteria in the urban Danube River (Novi Sad, Serbia) using a machine learning framework. **Methods**: Surface-water and wastewater samples were collected during summer and autumn 2024. Bacterial isolates were obtained through membrane filtration onto chromogenic media and identified using MALDI-TOF MS. Physicochemical parameters (including COD, BOD_5_, turbidity, pH, and temperature) were used as predictors in seven machine learning models (ANN, RF, SVM, XGB, MARS, TREE, NB). Model performance was assessed using AUC, accuracy, and error metrics. **Results**: Wastewater samples showed higher bacterial abundance and taxonomic richness than river surface-water samples, with frequent recovery of *Klebsiella pneumoniae*, *Escherichia coli*, *Aeromonas veronii*, and *Pseudomonas* spp. Tree-based models (RF, XGB) performed best. Organic pollution indicators, turbidity, pH, and water temperature were the most prominent factors. **Conclusions**: Wastewater-related pollution gradients, reflected by organic load parameters, turbidity, pH, and water temperature, were associated with the occurrence of selected bacterial taxa recovered on selective media. These associations were more pronounced in wastewater samples, while river surface-water samples showed lower abundance and taxonomic richness but still contained selected environmental and opportunistic taxa. Because antimicrobial susceptibility testing and molecular confirmation of resistance determinants were not performed, the findings should be interpreted as culture-based and exploratory. Machine learning approaches may support environmental screening and hypothesis generation for AMR-oriented surveillance, but future studies should include standardized phenotypic and molecular confirmation.

## 1. Introduction

Antimicrobial resistance (AMR) is a major global public health threat. In 2019, bacterial AMR caused 1.27 million deaths and was associated with 4.95 million deaths worldwide [1]. AMR is no longer confined to clinical settings. The environment is now recognized as a key reservoir and transmission pathway of resistance [2,3,4]. Surface waters play a central role in AMR dissemination. Rivers connect human activities with natural ecosystems. They integrate inputs from wastewater, hospital effluents, and agricultural runoff [5]. Aquatic environments, therefore, act as both sinks and sources of resistant bacteria [6]. Multidrug-resistant (MDR) bacteria are increasingly detected in river systems, especially those receiving wastewater discharges. Wastewater treatment plants are recognized as important reservoirs and release points for resistant bacteria and antibiotic resistance genes because conventional treatment processes may reduce but not completely eliminate these biological contaminants [7]. As a result, treated effluents can introduce resistant microorganisms and resistance determinants into receiving surface waters. In such environments, mobile genetic elements may facilitate the exchange of resistance genes between environmental bacteria and potentially pathogenic taxa. Resistant bacteria and antibiotic resistance genes have been detected not only in the water column but also in sediments and biofilms downstream of wastewater discharge points, supporting the role of river systems as environmental reservoirs and transport pathways for antimicrobial resistance [8,9]. This widespread distribution increases the risk of human and animal exposure, particularly when such waters are used for irrigation, recreation, or drinking water supply. These findings highlight the environmental dimension of antimicrobial resistance within a One Health framework [10]. The global spread of carbapenem resistance threatens the effectiveness of modern medicine. Increasing evidence shows that carbapenem-resistant bacteria are present in rivers, wastewater, and other environmental compartments [5]. These findings challenge the traditional view that carbapenem resistance is restricted to healthcare settings. River systems frequently contain both antibiotic residues and resistant microorganisms. Their occurrence is strongly linked to anthropogenic pressure [6]. However, the factors associated with their environmental distribution remain insufficiently understood. Antibiotics may exert selective pressure even at sub-inhibitory concentrations [11]. In contrast, resistance may persist independently of antibiotics due to co-selection mechanisms and ecological fitness advantages [12]. Environmental factors such as temperature, nutrient availability, hydrological conditions, and physicochemical parameters also play a key role [4]. These processes are complex, nonlinear, and context-dependent. Most existing studies apply conventional statistical approaches to analyze AMR–environment relationships. These methods often assume linearity and limited interactions. Environmental systems, however, exhibit strong nonlinear response. Machine learning approaches can better capture such complexity. Artificial Neural Networks (ANN), Random Forests (RF), Support Vector Machines (SVM), Extreme Gradient Boosting (XGB), Multivariate Adaptive Regression Splines (MARS), Decision Trees (TREE), and Naive Bayes (NB) have been widely applied in water quality prediction and environmental modeling [13,14,15]. These methods encompass both parametric and non-parametric frameworks, enabling the modeling of complex, nonlinear relationships and high-dimensional interactions that are commonly observed in environmental datasets [16]. For instance, ensemble-based techniques such as RF and XGB are particularly valued for their robustness to overfitting and their ability to capture variable importance [17], while kernel-based approaches like SVM are effective in handling nonlinearly separable data through transformation into higher-dimensional feature spaces [18].

Recent advances in computational capacity and data availability have further enhanced the applicability of these models, facilitating their integration with large-scale monitoring datasets and remote sensing inputs [19]. In parallel, methodological developments such as cross-validation, hyperparameter optimization, and feature selection have improved model generalizability and statistical reliability [20]. Importantly, recent studies have demonstrated the potential of these approaches for predicting the distribution and dynamics of AMR in aquatic systems [21,22]. These applications highlight the capacity of machine learning models to incorporate heterogeneous predictors, such as physicochemical parameters, land use characteristics, and microbial indicators, while accounting for spatial and temporal variability [23]. Consequently, these techniques are increasingly recognized as valuable tools for risk assessment, early warning systems, and decision support in water resource management and environmental health studies.

However, the application of ANN, RF, SVM, XGB, MARS, TREE, and NB in environmental AMR research remains limited. The Danube River is one of the largest river systems in Europe. It flows through densely populated and industrialized regions and is exposed to diverse anthropogenic pressures [24]. The urban section near Novi Sad (Serbia) represents a suitable model system for studying the combined effects of environmental factors on antibiotic occurrence and resistance patterns. The aim of this study was to evaluate the influence of environmental and physicochemical factors on the distribution of multidrug-resistant bacteria in Danube River water samples, as well as wastewater effluence in the Novi Sad city area across two different seasons (Summer and Autumn). ANN, RF, SVM, XGB, MARS, TREE, and NB were used to integrate microbiological, chemical, and environmental data. The results identify the most influential environmental factors of AMR and demonstrate the applicability of data-driven models for predicting AMR response in riverine systems. These findings contribute to improved environmental AMR surveillance and risk assessment within a One Health framework.

## 2. Results

### 2.1. Occurrence and Distribution of Bacterial Isolates Across Sampling Sites and Seasons

**Figure 1:**
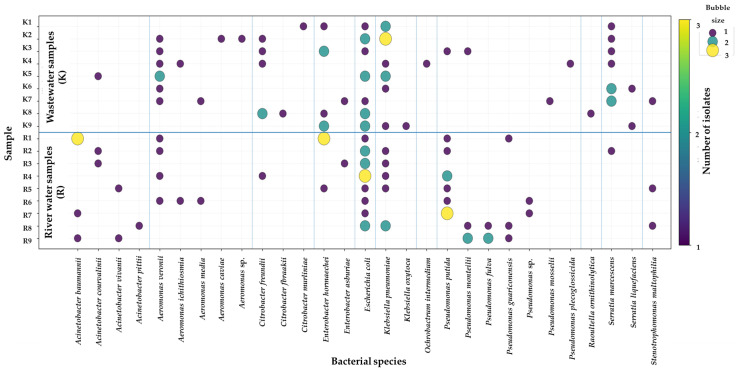
Bubble plot showing the number of isolates of each identified bacterial taxon recovered from each sampling site during the summer campaign in the Danube River area near Novi Sad. K-sites indicate wastewater samples, R-sites indicate river surface-water samples, and bubble size represents the number of isolates of the corresponding taxon at a given site.

**Figure 2:**
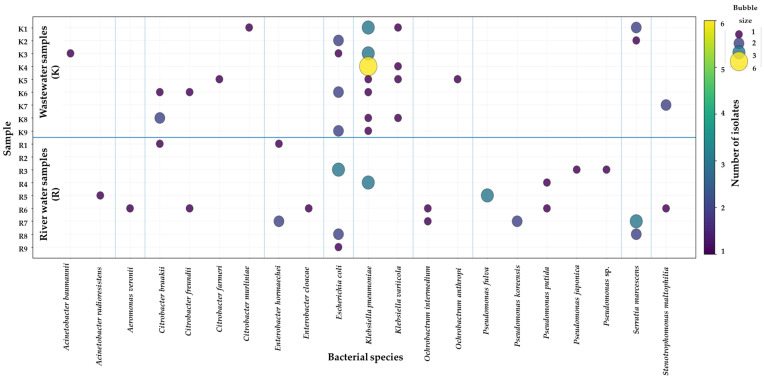
Bubble plot showing the number of isolates of each identified bacterial taxon recovered from each sampling site during the autumn campaign in the Danube River area near Novi Sad. K-sites indicate wastewater samples, R-sites indicate river surface-water samples, and bubble size represents the number of isolates of the corresponding taxon at a given site.

A total of 138 and 82 bacterial isolates were obtained from surface-water and wastewater samples collected during the summer (July 2024) and autumn (October 2024) campaigns, respectively. The recovered isolates comprised Gram-negative taxa belonging primarily to Enterobacterales, *Pseudomonas* spp., and *Aeromonas* spp. The most frequently recovered taxa included *Klebsiella pneumoniae*, *Escherichia coli*, *Aeromonas veronii*, and selected *Pseudomonas* spp. These taxa were more frequently recovered from wastewater samples than from river surface-water samples, while river samples showed lower isolate numbers and lower taxonomic richness. In brief, the isolates comprised a diverse set of Gram-negative bacteria, with dominance of genera belonging to Enterobacterales, *Pseudomonas* spp., and *Aeromonas* spp. The overall distribution of detected and identified isolates across sampling locations and seasons is presented in Figure 1 and Figure 2. A clear difference between wastewater (K sites) and river surface water (R sites) was observed. Wastewater samples exhibited both higher abundance (105 vs. 98 isolates in both seasons) and broader diversity of bacterial species (26 vs. 24 in both seasons) compared to river water samples. During the summer campaign (Figure 1), isolates were more widely distributed across both wastewater and river sites, with several species detected at multiple locations. Clinically relevant taxa, including *Klebsiella pneumoniae*, *Escherichia coli*, and *Enterobacter* spp., were frequently associated with wastewater samples, while environmental species such as *Aeromonas veronii* and *Pseudomonas* spp. were also detected in river water, indicating downstream dissemination from anthropogenic sources.

In contrast, the autumn campaign (Figure 2) showed a reduced number of isolates and a more localized distribution pattern. The majority of detected taxa were concentrated in wastewater samples, whereas river surface-water samples contained fewer isolates and lower taxonomic richness, defined here as the number of different bacterial taxa recovered from the analyzed samples. Across both seasons, several species were consistently detected in wastewater samples, highlighting their persistence in environments influenced by anthropogenic inputs. River samples, although less diverse, still contained opportunistic and environmental bacteria, confirming the role of the river system as a secondary reservoir and transport pathway. Due to insufficient sample size, taxa represented by fewer than two isolates across all sampling sites were excluded from further modeling, as this level of occurrence did not allow for reliable model training or robust classification. These included several low-frequency species such as *Acinetobacter* spp., *Aeromonas* spp., *Citrobacter* spp., and others, which were represented by only a few observations across sampling sites.

Across both sampling campaigns, a limited number of bacterial species accounted for the majority of detected isolates, indicating a non-uniform distribution pattern dominated by a few taxa. The most frequently detected species included *K. pneumoniae*, *E. coli*, *A. veronii*, and *Pseudomonas* spp. These taxa were consistently observed across multiple sampling sites, particularly in wastewater samples, suggesting their strong association with anthropogenic sources. In wastewater samples (K sites), *K. pneumoniae* and *E. coli* were among the most dominant species, often occurring with higher counts (≥2 isolates per sample), indicating continuous input from human-related sources. In contrast, river water samples (R sites) showed a lower abundance but still contained environmentally persistent species such as *A. veronii* and *Pseudomonas* spp., confirming downstream dissemination and environmental adaptation. Seasonal comparison revealed that the summer dataset was characterized by greater species richness and broader distribution, while the autumn dataset showed reduced diversity and more localized occurrence patterns, with dominance of a smaller number of species, primarily in wastewater samples.

It is very important to emphasize that isolates were recovered on selective/chromogenic media intended for the screening of bacterial groups of potential relevance to beta-lactam resistance, including ESBL- and carbapenemase-oriented screening media. However, recovery on these media was considered presumptive and was not interpreted as confirmatory evidence of ESBL or carbapenemase production. Confirmation of resistance phenotypes or resistance genes was beyond the scope of the present study and is identified as a key requirement for future work.

### 2.2. Performance of Machine Learning Models for Bacterial Distribution

The performance of machine learning models in predicting bacterial distribution was evaluated using a suite of classification algorithms, including artificial neural networks, random forest, support vector machines, extreme gradient boosting, multivariate adaptive regression splines, decision trees, and naïve Bayes. The machine learning models demonstrated the observed classification performance under the applied validation framework. However, because potential overlap of related isolates between training and testing subsets cannot be completely excluded, the reported performance metrics should be interpreted with caution and considered exploratory. Model performance was evaluated using multiple complementary metrics, including the area under the receiver operating characteristic curve (AUC), classification accuracy, sensitivity, and specificity. In addition, probability-based error metrics including RMSE (root mean square error), AARD (average absolute relative deviation), and SSE (sum of squared errors) and the coefficient of determination (R^2^) were calculated using predicted probabilities of class membership. These metrics were included to provide additional information on model calibration and the magnitude of prediction errors in probabilistic space, complementing standard classification performance measures. This multi-criteria approach enables a more comprehensive evaluation of both discriminative ability and the quality of probability estimates. Particular attention was given to model robustness and uncertainty, as reflected by confidence intervals of AUC values, ensuring a reliable comparison of algorithmic performance in capturing patterns of bacterial occurrence under varying environmental conditions. Due to insufficient sample size, a subset of identified microorganisms could not be included in the machine learning classification analysis. Specifically, species such as *Acinetobacter vivianii*, *A. pittii*, *A. radioresistens*, *A. ichthiosmia*, *A. media*, *A. caviae*, *Aeromonas* sp., *Citrobacter farmeri*, *C. murliniae*, *Enterobacter asburiae*, *E. cloacae*, *K. oxytoca*, *Ochrobactrum anthropi*, *Pseudomonas japonica*, *P. koreensis*, *P. mosselii*, *P. plecoglossicida*, *R. ornithinolytica*, and *S. liquefaciens* were represented by a limited number of observations. As a consequence, these classes were excluded from model development, as the available data were insufficient to support reliable training, validation, and performance evaluation of classification algorithms. The inclusion of such underrepresented classes would likely lead to model overfitting, unstable predictions, and unreliable performance metrics, particularly in terms of sensitivity, specificity, and calibration.

#### 2.2.1. Overall Model Behavior Across Bacterial Targets

The dataset used for classification model development comprised 18 bacterial taxa, including *A. baumannii*, *A. courvalinii*, *A. veronii*, *C. braakii*, *C. freundii*, *E. hormaechei*, *E. coli*, *K. pneumoniae*, *K. variicola*, *O. intermedium*, *P. fulva*, *P. guariconensis*, *P. monteilii*, *P. putida*, *Pseudomonas* sp., *S. marcescens*, and *S. maltophilia*. Predictor variables included both categorical and continuous environmental parameters, namely sampling site (K or R sites), sampling time (July or October), air temperature, relative air humidity, atmospheric pressure, water temperature, turbidity, pH, electroconductivity, chemical oxygen demand (COD), and biochemical parameter (BOD_5_). These variables were selected to capture the physicochemical and temporal conditions potentially influencing bacterial distribution and were used as input features for the development of a multiclass classification model. The gained model performance varied substantially across bacterial taxa, reflecting strong species-dependent predictability and pronounced effects of class imbalance. The results were graphically presented in Figure 1 and Figure 2, and also in Supplementary Materials S1. Supplementary Materials S2 provides a comprehensive set of tables that detail the performance and reliability of the machine learning models applied in this study for predicting microbial occurrence in aquatic environments. These tables are intended to complement the main text by offering expanded quantitative results, enabling a more thorough evaluation of model behavior across different data inputs and performance metrics. Specifically, the first group of tables summarizes the predictive performance of the implemented models, ANN, RF, SVM, XGB, MARS, TREE, and NB, using integrated microbiological, chemical, and environmental datasets. Standard regression and classification performance indicators are reported, allowing for a comparative assessment of model accuracy and generalization capacity.

The second set of tables focuses on classification performance, presenting metrics such as accuracy, sensitivity, specificity, precision, and F1-score. These measures provide insight into the models’ ability to correctly identify the presence or absence of microbial contamination, which is particularly relevant for environmental monitoring and risk assessment applications. The third group of tables reports the distribution of prediction confidence levels alongside corresponding Brier scores, offering an evaluation of probabilistic prediction quality and calibration. By quantifying the agreement between predicted probabilities and observed outcomes, these results contribute to a deeper understanding of model uncertainty and reliability. Together, Supplementary Materials S1 and S2 enhance transparency and reproducibility, and support a rigorous comparison of machine learning approaches for microbial prediction across varying environmental conditions.

ROC curves were used to evaluate and compare the classification performance of the applied machine learning models. ROC diagrams illustrate the trade-off between sensitivity (true positive rate) and 1–specificity (false positive rate) across different probability thresholds, providing a threshold-independent assessment of model discrimination ability. The area under the ROC curve (AUC) serves as a summary metric, where values closer to 1 indicate better model performance in distinguishing between presence and absence of the target microorganism.

Discriminative performance (AUC) ranged from poor to moderately high depending on the target, with consistently higher values observed for *K. pneumoniae*, *K. variicola*, *A. veronii*, and several *Pseudomonas* spp. (often AUC > 0.80), while lower and unstable performance was evident for *C. freundii*, *E. hormaechei*, and *E. coli* (AUC frequently near or below 0.60). In general, accuracy values appeared moderately high across models (often 0.75–0.92), but this was not consistently supported by AUC or R^2^, indicating that accuracy alone overestimated predictive performance due to class imbalance. Sensitivity was typically high, whereas specificity was frequently very low or near zero for many models, confirming a strong bias toward positive class prediction and a high false-positive rate; see Supplementary Materials S2.

Among algorithms, tree-based ensemble methods, particularly random forest (RF) and extreme gradient boosting (XGB), showed the most stable and reliable performance across taxa, with relatively high AUC values and lower prediction error (RMSE, SSE) compared to linear or probabilistic approaches; see Supplementary Materials S2. Decision trees occasionally achieved high AUC and sensitivity, but with reduced stability and weaker calibration. Support vector machines (SVMs) performed inconsistently, ranging from strong discrimination in some taxa to weak or unstable results in others. Artificial neural networks (ANNs) showed moderate but generally less stable performance, while naïve Bayes (NB) and MARS were highly sensitive to dataset structure and class imbalance, often producing inflated or unstable error metrics and poor R^2^ values (frequently negative).

In terms of calibration and predictive reliability, RF and XGB demonstrated the most consistent balance between discrimination and error minimization, particularly for *Klebsiella*, *Pseudomonas*, and *S. maltophilia*. However, across most taxa, R^2^ values were frequently low or negative, indicating limited explanatory power of the models in a regression-equivalent sense and reinforcing the classification-driven nature of the task. Overall, model verification highlights that reliable prediction of bacterial occurrence was achievable only for a subset of taxa, while many species exhibited unstable or biased classification performance driven primarily by data imbalance and overlapping environmental signatures; see Supplementary Materials S2.

#### 2.2.2. Best-Performing Bacterial Targets

**Figure 3:**
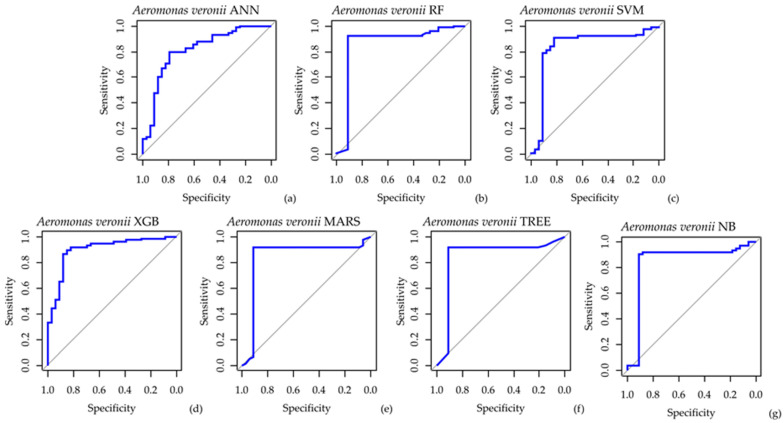
ROC curves for classification of *A. veronii* using (**a**) ANN, (**b**) RF, (**c**) SVM, (**d**) XGB, (**e**) MARS, (**f**) TREE, and (**g**) NB models.

Among all evaluated taxa, *A. veronii* demonstrated the strongest and most stable predictive performance (Supplementary Materials S2). Although XGB achieved the highest discrimination capacity (AUC = 0.899; CI: 0.832–0.966), model performance was interpreted not only according to AUC values, but also considering sensitivity, specificity, accuracy, and error metrics, because high AUC values may coexist with imbalanced class prediction. TREE demonstrated the most balanced overall performance, achieving high classification accuracy (0.917) together with balanced sensitivity and specificity values (0.92 and 0.91, respectively), as well as the lowest error metrics (RMSE = 0.286; SSE = 8.84), indicating consistent predictive and well-calibrated classification performance. RF also showed strong discrimination ability (AUC = 0.858; CI: 0.757–0.959), while XGB maintained high sensitivity (0.92) and favorable error control (RMSE = 0.321; AARD = 21.02), supporting its suitability for detecting nonlinear environmental relationships. The ROC curves presented in Figure 3 illustrate differences among ANN, RF, SVM, XGB, MARS, TREE, and NB models in their ability to classify microbial occurrence under varying environmental conditions. Variations in curve shape, sensitivity, and specificity indicate differences in model discrimination capacity and classification balance across presence and absence classes.

**Figure 4:**
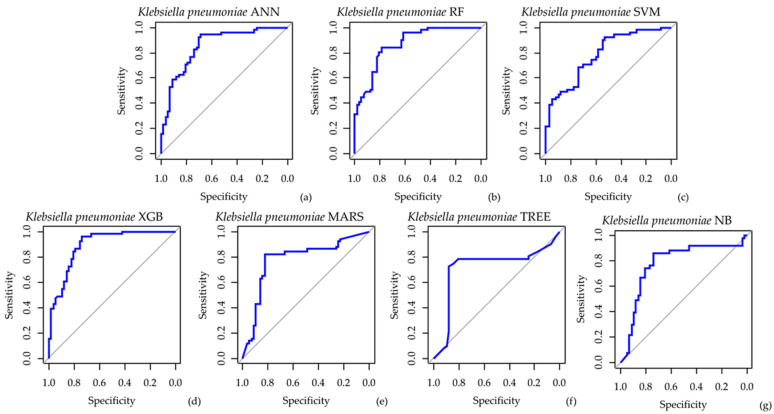
ROC curves for classification of *K. pneumoniae* using (**a**) ANN, (**b**) RF, (**c**) SVM, (**d**) XGB, (**e**) MARS, (**f**) TREE, and (**g**) NB models.

*K. pneumoniae* represented the second most reliable prediction target, with consistently high AUC values across models. The best performance was obtained by XGB (AUC = 0.892; CI: 0.832–0.952), followed by RF (AUC = 0.869; CI: 0.804–0.934) and ANN (AUC = 0.853; CI: 0.782–0.925). Although classification accuracy was moderate (0.75–0.80), regression-based indicators showed clearer separation, with XGB achieving the lowest RMSE (0.387) and improved explanatory power (R^2^ = 0.399). TREE also provided stable performance (AUC = 0.717; accuracy = 0.796), suggesting moderate but consistent predictability. The ROC diagram (Figure 4) compares the predictive performance of ML models for *K. pneumoniae*, with differences in curve shape and AUC indicating variation in classification accuracy across models.

**Figure 5:**
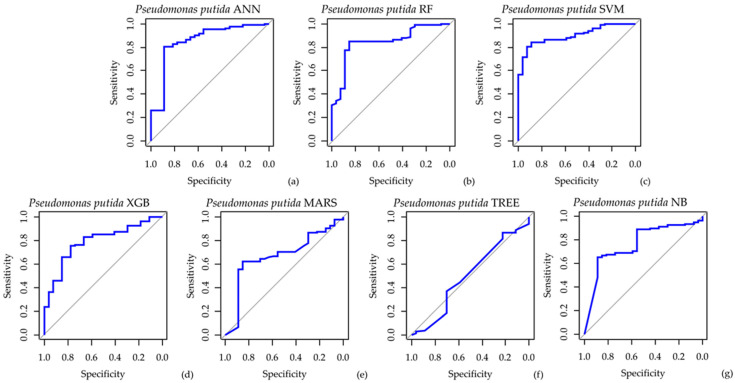
ROC curves for classification of *P. putida* using (**a**) ANN, (**b**) RF, (**c**) SVM, (**d**) XGB, (**e**) MARS, (**f**) TREE, and (**g**) NB models.

*Pseudomonas putida* represented intermediate but ecologically meaningful case. For *P. putida*, the strongest discrimination was achieved by SVM (AUC = 0.900; CI: 0.842–0.957), followed by ANN (AUC = 0.853) and RF (AUC = 0.844). However, despite a high AUC, calibration remained weaker, with RMSE ranging from 0.36 to 0.53 and noticeable variability in specificity, suggesting model sensitivity to class imbalance. The ROC diagram (Figure 5) presents the ROC diagram of machine learning models for *P. putida*, where variations in curve shape and AUC values indicate differences in classification accuracy among models.

In contrast, *E. coli* represented a clearly challenging target with limited predictive structure. The highest AUC values remained low (MARS = 0.699; RF = 0.675; XGB = 0.656), while ANN performed poorly (AUC = 0.482; R^2^ = −0.689). Accuracy ranged only from 0.56 to 0.72, and error values were substantially higher than for other taxa (RMSE up to 0.634; SSE = 43.35), indicating weak model calibration and poor environmental separability.

The results highlight a clear gradient in predictability across bacterial taxa, ranging from highly structured and well-modeled species (*A. veronii*, *K. pneumoniae*) to intermediate case (*P. putida*), and finally to poorly predictable taxa (*E. coli*). Tree-based ensemble methods, particularly XGB and TREE, consistently provided the best combination of high AUC (up to ~0.90), strong sensitivity (often ≥0.92), and reduced error (RMSE down to ~0.28–0.32), confirming their superior performance in capturing complex nonlinear environmental relationships and mitigating class imbalance effects.

#### 2.2.3. Challenging Targets and Limitations

In contrast to the best-performing taxa, *C. freundii*, *S. marcescens*, and *E. hormaechei* represent clearly weaker and less stable modeling cases, highlighting important limitations of the developed classification framework. For *C. freundii*, predictive performance was generally inconsistent across all models. AUC values ranged from very poor to moderate (0.340–0.765), with the lowest discrimination observed for the decision tree model (AUC = 0.340; CI: 0.272–0.409), indicating almost random classification behavior. Even the best-performing model (SVM) achieved only moderate discrimination (AUC = 0.765; CI: 0.653–0.877), but this was not supported by stable calibration, as reflected in negative R^2^ values across all models (down to −0.876 for NB and −0.497 for ANN). Despite relatively high accuracy values (up to 0.81), this was driven by class imbalance rather than true predictive skill, as confirmed by extremely low specificity (often <0.15 and in several cases near zero). Error metrics further confirm instability, with RMSE reaching 0.542 and AARD exceeding 33% in several models, indicating poor quantitative agreement and weak generalizability. Similarly, *Serratia marcescens* showed uniformly modest and unstable predictive performance. AUC values remained low across all models (0.562–0.694), with no algorithm achieving strong discrimination. The highest AUC was observed for NB (0.694), followed by RF (0.683) and XGB (0.675), but these values still indicate only limited separability from environmental predictors. Classification accuracy ranged between 0.62 and 0.74, but again without meaningful specificity (mostly <0.35), suggesting a strong bias toward overprediction of presence. Calibration performance was weak, with negative or near-zero R^2^ values for most models (down to −0.418 for MARS), and relatively high error levels (RMSE up to 0.544; SSE > 30 in several cases). AARD values also remained elevated (up to 41.28%), confirming low predictive reliability. *E. hormaechei* further illustrates one of the weakest and least reliable prediction cases in the dataset, showing overall poor discriminative and calibration performance across all evaluated models. AUC values ranged from very low to moderate (0.313–0.660), with the lowest performance observed for the decision tree (AUC = 0.313; CI: 0.211–0.415) and MARS (AUC = 0.354; CI: 0.251–0.458), indicating near-random classification behavior. Even the best-performing model (SVM) achieved only moderate discrimination (AUC = 0.660; CI: 0.534–0.786), which was not accompanied by reliable calibration. These taxa demonstrate that not all bacterial species can be reliably modeled using the selected environmental predictors. The consistently low AUC values, poor calibration (negative R^2^), and severely reduced specificity indicate that these organisms either have weak or indirect relationships with measured environmental variables or are influenced by unmeasured ecological factors. These results emphasize the limitations of the current feature set and highlight the need for additional biological or molecular predictors to improve model performance for environmentally less structured taxa.

#### 2.2.4. Environmental Factors Associated with Bacterial Occurrence

Supplementary Materials S3 summarizes the main predictors driving microbial quantity across the applied machine learning models (RF, SVM, ANN, and XGB). Across all models and microbial targets, the most influential factors consistently include organic pollution indicators (COD—chemical oxygen demand proxy, BOD_5_—biochemical oxygen demand), temperature (water and air), turbidity, pH, relative air humidity, and site- and time-specific variables, highlighting the combined importance of physicochemical conditions, seasonal effects, and spatial variability in shaping microbial occurrence. Ensemble models (RF, XGB) highlighted these factors through variable importance measures, while SVM and ANN captured their combined nonlinear effects. Despite differences in model structure, there is consistent agreement on the key environmental factors controlling microbial response. Across all 18 bacterial targets, the machine learning models consistently indicate that bacterial occurrence is primarily governed by water-quality (physicochemical) variables rather than meteorological forcing, although the strength and hierarchy of predictors vary substantially between taxa. The most influential and repeatedly dominant variables across RF, SVM, ANN, and XGBoost models are COD, BOD_5_, turbidity, pH, and water temperature, with additional but secondary contributions from electroconductivity. For example, COD reaches importance values as high as 0.71 for *P. putida* (XGB), 0.67 for *Pseudomonas* sp., and 0.47 for *S. marcescens*, and remains consistently among the top predictors across most taxa (typically in the range ~0.13–0.36 in RF and ANN models). Similarly, BOD_5_ shows strong influence for *A. baumannii* (~0.11 RF), *C. braakii* (~0.23 RF), and *E. coli* (~0.13–0.21) across models, indicating a strong link between biodegradable organic load and bacterial presence. Turbidity emerges as another strong predictor, particularly for *C. freundii* (~0.24 RF), *E. coli* (~0.16 RF, ~0.56 XGB), *S. marcescens* (~0.07 RF but higher in SVM/ANN), and *P. putida* (~0.10 RF), suggesting that suspended particulate matter strongly mediates microbial habitats. Water temperature is especially important for thermally sensitive taxa such as *A. veronii* (up to ~0.34 RF and strong XGB contributions), *E. hormaechei* (~0.31 RF), and *K. pneumoniae* (~0.29 XGB), indicating a clear thermal control on growth dynamics. pH also shows strong nonlinear importance, particularly in SVM and XGB models, reaching values of ~0.46 for *C. braakii*, ~0.37 for *P. monteilii*, ~0.34 for *K. pneumoniae*, and ~0.28 for *A. veronii*, suggesting that acidity/alkalinity is a key structuring factor for several taxa. In contrast, meteorological variables such as air temperature, relative humidity, and pressure generally show weaker and less consistent influence, although air temperature becomes important for selected species such as *A. veronii* (up to ~0.34 RF and ~0.65 XGB) and *P. putida* (moderate contributions across models). Notably, categorical variables capturing site and season (Time) also exhibit substantial explanatory power in many cases, often rivaling physicochemical predictors; for instance, Time (July) and Time (October) appear as leading factor associated with *A. veronii*, *C. braakii*, and *E. hormaechei*, while spatial effects (Site (K), Site (R)) are particularly strong for *Klebsiella* spp. and *Pseudomonas* spp., indicating pronounced spatial heterogeneity in microbial distribution.

The results demonstrate a clear hierarchy of factors: (1) organic pollution and nutrient load variables (COD, BOD_5_), (2) hydrological quality indicators (turbidity, pH, water temperature), (3) spatial–temporal structure (site and season), and (4) meteorological conditions as secondary modulators, with notable inter-bacterial differences reflecting distinct ecological niches, ranging from strong pollution-associated responders (*Citrobacter* spp., *Enterobacter* spp.) to more environmentally structured generalists (*Pseudomonas* spp., *E. coli*), confirming that microbial response in the system are primarily controlled by water quality gradients rather than atmospheric forcing.

## 3. Discussion

### 3.1. Occurrence and Distribution of Bacterial Isolates Across Sampling Sites and Seasons

The present study supports the hypothesis that wastewater-associated environmental gradients play a major role in structuring the occurrence of selected clinically relevant and opportunistic bacteria in the urban Danube system. Across the two sampling campaigns, wastewater sites consistently showed higher abundance and broader diversity of recovered isolates than river surface-water sites, while the summer campaign yielded more isolates and a wider spatial distribution than the autumn campaign. Taken together, these findings suggest that bacterial occurrence in this system is shaped by a combination of source intensity, habitat suitability, and species-specific ecological plasticity rather than by a single uniform environmental factor. The wastewater-to-river gradient observed here is consistent with recent evidence that municipal wastewater is the most influential factor associated with AMR-related contamination in surface waters. In a whole-river Danube study, Schachner-Groehs et al. [25] linked ARG patterns primarily to human fecal pollution from municipal wastewater and detected ESBL- and carbapenemase-related genes at hotspot locations. Likewise, Mollenkopf et al. [26] found carbapenemase-producing Enterobacterales throughout a wastewater-treatment and downstream watershed continuum over one year, and Ruiz et al. [27] showed that urban wastewater overflows and downstream river sections can carry viable ESBL- and carbapenemase-producing bacteria for kilometers below impacted areas. Against this background, the stronger signal at wastewater sites in the present study is best interpreted as evidence of sustained anthropogenic loading and downstream dissemination rather than isolated contamination events. Within the limitations of two sampling campaigns, the stronger signal observed at wastewater sites in this study is therefore interpreted as a reflection of localized anthropogenic loading rather than evidence of broader or long-term ecological processes. Further longitudinal sampling would be required to assess temporal stability and ecological factors of these patterns.

At the taxon level, the recurrent detection of *K. pneumoniae* and *E. coli* in wastewater samples fits their close association with human waste streams, whereas the persistence of *A. veronii* and *Pseudomonas* spp. in river samples suggests that environmentally adapted organisms may remain detectable after downstream transport. This interpretation is strengthened by recent work showing that environmental compartments can function as secondary reservoirs rather than passive recipients alone. Leopold et al. [28] reported that antibiotic resistance ratios were higher in Danube biofilm than in overlying water and detected ESBL-producing *E. coli* in biofilm but not in river water, implying that attached communities may retain and stabilize resistance patterns. Similarly, Martínez-Ruiz et al. [29] detected carbapenem-relevant environmental taxa, including *Aeromonas* spp. and environmental *Pseudomonas* spp., in recycled wastewater and argued for routine monitoring beyond standard fecal indicators. The observed differences between the two sampling campaigns suggest temporal variability, but they do not allow firm conclusions regarding seasonality or stable ecological niches. With only two campaigns, the present dataset supports a seasonal association more strongly than a definitive seasonal mechanism. Even so, this interpretation aligns with broader environmental AMR work. Ramessar et al. [30] found strong seasonal and spatial effects on physicochemical parameters, ARB, and ARGs across multiple wastewater plants and receiving waters, while Nishimura et al. [31] showed that year-round variation in resistant bacteria can track environmental conditions and source activity. Danube-wide monitoring has similarly shown that temporal hotspots can occur even within the same river system. For this reason, the safest conclusion is that summer conditions in the present study likely favored detection, persistence, or transport of several taxa, but denser temporal sampling is needed to disentangle temperature, dilution, flow regime, rainfall, and episodic sewage inputs.

### 3.2. Performance of Machine Learning Models for Bacterial Distribution

A central result of this study is that bacterial occurrence was not equally predictable across taxa. The strongest predictive structure was observed for *A. veronii* and *K. pneumoniae*, whereas *E. coli*, *C. freundii*, and *E. hormaechei* were substantially harder to classify reliably. This gradient in predictability is informative in itself: it implies that some taxa track measured aquatic conditions more closely than others. Methodologically, the present comparison also shows that apparently high accuracy can be misleading in imbalanced datasets when specificity is poor. That point deserves explicit discussion, because the manuscript’s best contribution is not only that some classifiers performed better, but that the multi-metric evaluation exposed when performance was inflated by class distribution rather than genuine discriminative skill. The superior overall performance of RF and XGB in this study is consistent with the recent environmental AMR modeling literature. Jiang et al. showed that an integrated machine learning framework using accessible environmental variables could rapidly quantify AMR burden with high performance, while Yang et al. [32] found that XGBoost performed best for resistome risk prediction across diverse water environments. A recent systematic review by Foroughi et al. [33] further concluded that machine learning applications in water and wastewater AMR remain relatively sparse, are dominated by supervised shallow-learning approaches, and still suffer from limited standardization of evaluation criteria. In that context, the present manuscript adds useful evidence from a real river–wastewater interface by showing that ensemble methods are not universally “best” in an abstract sense, but are especially effective when ecological patterns are nonlinear and taxon-specific. The variable importance patterns also support a coherent ecological interpretation. Organic load indicators (COD/BOD_5_, corresponding to COD/BOD_5_-type measures), turbidity, pH, and water temperature were more influential than broad meteorological descriptors, suggesting that bacteria responded primarily to the immediate aquatic habitat and pollution gradient rather than to atmospheric conditions alone. This interpretation is plausible for several reasons: organic load indexes wastewater influence and nutrient availability; turbidity often reflects suspended particles that can transport and protect bacteria; pH and temperature affect growth, persistence, and competition; and site/season likely encode source proximity and hydrological context. Recent Danube-scale and wastewater-treatment studies similarly found that wastewater-associated fecal pollution and physicochemical conditions are major determinants of AMR patterns [25,34]. A reasonable interpretation is therefore that *A. veronii* and *K. pneumoniae* were more predictable because they were more tightly coupled to measurable source and habitat gradients, whereas *E. coli* may have reflected multiple overlapping inputs and more transient transport processes. That last point should be presented as a plausible mechanism rather than a demonstrated one.

The observed variability in model performance across bacterial taxa reflects fundamental differences in their ecology, environmental adaptability, and source attribution rather than purely statistical behavior [35]. The higher predictive performance achieved for certain taxa (e.g., *K. pneumoniae*, *A. veronii*, *Pseudomonas* spp.) suggests that these organisms are strongly structured by measurable environmental gradients, particularly those associated with wastewater influence and organic pollution. These bacteria are well known for their ecological plasticity and ability to persist under fluctuating physicochemical conditions [36,37,38], which makes their occurrence more consistently linked to variables such as organic load (COD/BOD_5_), turbidity, and temperature. In practical terms, this means that their presence in aquatic systems is not random but instead reflects relatively stable ecological niches shaped by anthropogenic inputs. In contrast, taxa that exhibited poor or unstable model performance (e.g., *E. coli*, *Enterobacter* spp., *Citrobacter* spp.) likely represent ecologically more complex or transient populations. For instance, *E. coli* is widely used as a fecal indicator organism, but its environmental response is influenced by multiple overlapping sources (human, animal, environmental reservoirs), as well as short-term hydrological events and dilution effects [39]. As a result, its occurrence is less tightly coupled to the measured environmental parameters, leading to weaker model predictability despite its frequent detection. The exclusion of low-frequency taxa from modeling is also microbiologically meaningful. Rarely detected species may represent sporadic contamination events rather than stable populations, niche specialists with narrow ecological requirements not captured by the measured variables, or under-sampled taxa whose apparent rarity reflects methodological limitations rather than true absence [40]. Including such taxa in predictive models would not only reduce statistical reliability but would also obscure biologically relevant patterns by introducing noise from ecologically unstructured or insufficiently characterized populations. Importantly, the need for balanced datasets in machine learning aligns with a key microbiological principle: reliable ecological inference requires sufficient representation of each taxon across environmental gradients [41]. Without this, it becomes difficult to distinguish between true ecological absence and sampling bias. Overall, the modeling results indicate that bacterial taxa in the studied system can be broadly divided into (i) environmentally structured taxa (predictable, pollution-associated, persistent) and (ii) ecologically diffuse or transient taxa (poorly predictable, multi-source, event-driven). This distinction has direct implications for AMR surveillance. It suggests that not all bacterial indicators are equally suitable for environmental monitoring, and that combining multiple taxa with different ecological behaviors may provide a more comprehensive picture of AMR dissemination in aquatic systems.

Additionally, the observed heterogeneity in model performance across bacterial taxa reflects fundamental ecological differences in environmental adaptation, niche specificity, and source dynamics rather than purely statistical variability. The strong predictability observed for taxa such as *K. pneumoniae*, *K. variicola*, *A. veronii*, and several *Pseudomonas* spp. (AUC > 0.80) indicates that these organisms are closely linked to stable and measurable environmental gradients, particularly those associated with wastewater inputs and organic pollution. In ecological terms, they behave as environmentally structured or pollution-responsive taxa, whose distribution reflects relatively predictable habitat conditions [42]. *Enterobacter* and *Citrobacter* species may occupy transitional ecological niches, responding to both anthropogenic inputs and natural environmental reservoirs, which reduces their predictability [43,44,45]. The imbalance between high sensitivity and low specificity observed across models also has a microbiological explanation. It suggests that many taxa are widely distributed at low abundance or intermittently present, leading to frequent false-positive predictions. This reflects the reality that aquatic systems often contain background microbial populations with overlapping ecological signatures, making precise discrimination between presence and absence inherently difficult. Intermediate cases, such as *P. putida*, are particularly informative. These taxa show partial environmental structuring, where certain conditions (e.g., organic load or temperature) influence their occurrence, but not strongly enough to produce fully stable predictive patterns. This positions them as ecological generalists, capable of surviving across a wide range of conditions, but without a single most influential environmental factor. The superior performance of tree-based ensemble models (RF and XGB) further supports the microbiological interpretation that bacterial distribution is governed by nonlinear and interacting environmental factors. Microbial responses to environmental gradients are rarely linear; instead, they involve threshold effects, synergistic interactions (e.g., between nutrients and temperature), and niche competition, which are better captured by these models. Finally, the observed gradient in predictability, from highly predictable (*A. veronii*, *K. pneumoniae*), through intermediate (*P. putida*), to poorly predictable taxa (*E. coli*), can be interpreted as a reflection of ecological organization within the microbial community: (i) highly predictable taxa (strongly environment-driven, pollution-associated, stable niches); (ii) moderately predictable taxa (generalists with partial environmental dependence); (iii) poorly predictable taxa (transient, multi-source, or event-driven populations). This classification has important implications for AMR monitoring. It suggests that reliable environmental indicators should preferentially include taxa with strong ecological coupling to measurable environmental gradients, while commonly used indicators such as *E. coli* may not adequately capture the full complexity of AMR dissemination in aquatic systems.

A limitation of this study is that grouped/block validation was not performed to ensure complete separation of isolates originating from the same sample during model training and testing. Consequently, the possibility of partial information leakage cannot be entirely excluded, potentially leading to optimistic estimates of model performance. Therefore, the machine learning findings should be interpreted as exploratory and hypothesis-generating rather than confirmatory. Future studies should incorporate grouped validation strategies and independent external datasets to provide more robust assessment of model generalizability.

### 3.3. Strengths, Limitations, and Implications

This study has several strengths. It integrates culture-based recovery using chromogenic media for carbapenem-resistant and ESBL-positive Gram-negative bacteria, MALDI-TOF identification, physicochemical characterization, spatial comparison between wastewater and river sites, and a multi-algorithm modeling framework. It also evaluates model behavior using AUC, error metrics, and sensitivity/specificity rather than accuracy alone, which is essential in imbalanced ecological datasets. At the same time, the limitations should be stated explicitly. The analysis is based on two sampling campaigns and a modest number of observations distributed across many taxa, which created severe class imbalance and forced the exclusion of low-frequency classes. The visible methods also indicate selective culture and species identification, but do not yet show isolate-level susceptibility confirmation, targeted ESBL and carbapenemase genotyping, or whole-genome sequencing in the section provided here. In addition, the current feature set does not include hydrological discharge, rainfall events, sediment and biofilm reservoirs, antibiotic residues, heavy metals, or wastewater-infrastructure performance, all of which have been implicated in related riverine AMR studies. Finally, feature-importance outputs should not be treated as proof of direct causation.

On the other hand, this study has several important limitations that should be considered when interpreting the findings. First, the microbiological approach was culture-based and relied on selective/chromogenic media followed by MALDI-TOF MS identification. Although this approach is useful for environmental screening and recovery of clinically relevant bacterial taxa, it does not provide confirmatory evidence of antimicrobial resistance. In particular, antimicrobial susceptibility profiles, ESBL or carbapenemase production, and resistance genes were not confirmed by standardized phenotypic assays, qPCR, or sequencing. Therefore, the present results should not be interpreted as a definitive characterization of MDR or AMR patterns in the Danube River. Instead, they provide an exploratory assessment of the occurrence of selected bacterial taxa recovered under selective culture conditions and their association with wastewater-related environmental parameters. Future studies should integrate culture-based isolation with antimicrobial susceptibility testing, molecular detection of resistance genes, metagenomic or qPCR-based approaches, and measurements of antibiotic residues and other co-selective agents, such as heavy metals, to provide a more complete assessment of environmental AMR response. Additionally, the present findings support prioritizing wastewater discharges and adjacent river reaches as surveillance nodes in the urban Danube system. They further suggest that monitoring should not rely on *E. coli* alone, because *Klebsiella* spp., *Aeromonas* spp., and *Pseudomonas* spp. may capture different ecological aspects of AMR dissemination. A second implication is methodological. Tree-based machine learning models appear useful as screening tools for identifying high-risk taxa or conditions, but they should complement rather than replace laboratory confirmation. More broadly, the study aligns with the current One Health view that wastewater surveillance is an important interface linking human, environmental, and potentially food-chain exposure routes. Future research should therefore move in five concrete directions: first, year-round repeated upstream-to-downstream transects, ideally monthly or biweekly and including rainfall-event sampling, to resolve temporal dynamics; second, parallel analysis of influent, effluent, river water, sediment, and biofilm to capture reservoirs that water-only sampling may miss; third, isolate-level susceptibility testing plus qPCR/ddPCR or whole-genome sequencing for major ESBL and carbapenemase determinants; fourth, integration of antibiotic residues, metals, pesticides, and hydrological variables into the predictor set; and fifth, externally validated and better-calibrated classification frameworks, including class-balancing strategies and prospective validation on a new sampling year. Where infrastructure changes are planned, a before–after–control–impact design around wastewater-treatment upgrades or overflow control would be especially valuable.

## 4. Materials and Methods

### 4.1. Sampling

Sampling was conducted within the framework of the TRACE project (Grant No. 7042, WP4) in the metropolitan area of Novi Sad. Surface-water and wastewater samples were collected during two sampling campaigns performed in July (1–15 July 2024) and October (1–15 October 2024), following a predefined sampling plan. Sampling activities were carried out by the Public Utility Company “Vodovod i kanalizacija” Novi Sad. Surface-water and wastewater samples were collected according to standard procedures for the examination of water and wastewater, following APHA-based guidance for environmental water sampling. Samples were collected in sterile containers, transported to the laboratory under cooled conditions, and processed within the same working day. For each sampling site, the sample type, sampling campaign, and environmental conditions were recorded. The sampling design included wastewater-impacted sites and river surface-water sites in the urban Danube River section near Novi Sad, enabling comparison between wastewater-associated and riverine compartments. All samples were delivered to the laboratories of the Faculty of Medicine, University of Belgrade, for further microbiological characterization.

### 4.2. Environmental Parameters and Physicochemical Analysis

At each sampling location, a set of environmental and physicochemical parameters was recorded and used as input variables (environmental factors) for further analysis and modeling. These parameters were selected to capture both meteorological conditions and water quality characteristics that may influence the distribution of antibiotic resistance and antibiotic concentrations. Meteorological parameters, including air temperature (°C), relative humidity (%), and barometric pressure (hPa), were obtained at the time of sampling using validated online meteorological databases. In parallel, in situ and laboratory-based measurements of water quality were performed.

Physicochemical characterization included water temperature, pH, electroconductivity, turbidity, chemical oxygen demand (COD), and biochemical oxygen demand after five days (BOD_5_). Temperature, pH, electroconductivity, and turbidity were measured using calibrated field or laboratory instruments according to the manufacturer’s instructions and standard water-analysis procedures. COD and BOD_5_ were determined using standard methods for wastewater and surface-water analysis. These parameters were selected because they reflect organic load, physicochemical water quality, and wastewater-related environmental gradients potentially associated with bacterial occurrence. In brief, physicochemical parameters of water included water temperature (°C), turbidity (NTU), pH, and electrical conductivity (µS/cm). In addition, organic pollution indicators were determined, including chemical oxygen demand (COD, expressed as mg O_2_/L, dichromate method) and biochemical oxygen demand over 5 days (BOD_5_, mg O_2_/L). All analyses were conducted by the accredited laboratory of the Public Utility Company “Vodovod i kanalizacija” Novi Sad using standardized methods, including Standard Methods for the Examination of Water and Wastewater (SMEWW) and EPA protocols. Specifically, water temperature and conductivity were determined according to SMEWW methods 2550B and 2510B, pH according to SMEWW 4500-H^+^, and turbidity using EPA method 180.1. COD and BOD_5_ were determined using validated internal methods aligned with regulatory requirements. The obtained dataset of environmental parameters was used as input variables in subsequent statistical and machine learning analyses to evaluate their influence on the occurrence of carbapenem-resistant Enterobacterales and antibiotic concentrations.

### 4.3. Sample Processing, Bacterial Isolation and Identification

For microbiological analysis, 500 mL of each water sample was subjected to membrane filtration using sterile membrane filters (0.45 μm pore size). After filtration, membranes were aseptically transferred into sterile buffer solution and resuspended by vortexing to detach retained microorganisms. Aliquots of the resulting suspension were then plated onto selective chromogenic media for the isolation of carbapenem-resistant and extended-spectrum β-lactamase (ESBL)-producing bacteria, including ChromID^®^ CARBASMART and ChromID^®^ ESBL agar (bioMérieux, Marcy-l’Étoile, France). Plates were incubated aerobically at 37 °C for 18–24 h. After incubation, morphologically distinct colonies were selected based on their characteristic coloration and growth patterns on the respective chromogenic media. Selected isolates were identified using matrix-assisted laser desorption/ionization time-of-flight mass spectrometry (MALDI-TOF MS) (Microflex^®^ LRF, Bruker, Germany), following the manufacturer’s instructions. Identification was accepted at the species level according to the instrument’s score criteria. Selective/chromogenic media were used to recover bacterial groups of potential relevance for antimicrobial resistance-oriented environmental screening. However, growth on these media was interpreted only as presumptive selection and not as confirmatory evidence of antimicrobial resistance. No standardized antimicrobial susceptibility testing, ESBL/carbapenemase phenotypic confirmation, qPCR-based detection of antimicrobial resistance genes, or sequencing-based resistome analysis was performed in the present study. Therefore, the term “presumptive resistant isolates” is used cautiously, and the machine learning analysis was based on the occurrence of identified bacterial taxa rather than confirmed resistance phenotypes or genotypes.

### 4.4. Assessment of Environmental Factors Affecting MDR Bacteria

For machine learning analysis, observations were structured at the isolate/taxon-occurrence level, whereas physicochemical predictors were measured at the sample level. Consequently, isolates recovered from the same sample shared identical environmental predictor values. This data structure may introduce partial non-independence among observations and was considered when interpreting model outputs. Therefore, the models were used as exploratory tools for identifying associations between sample-level environmental conditions and recovered bacterial taxa, rather than as confirmatory predictive models.

#### 4.4.1. Data Preprocessing

The dataset comprised multiple identified bacterial species. Prior to modeling, the dataset was subjected to preprocessing. Missing values, if present, were handled using appropriate imputation strategies. All continuous variables were normalized using min–max scaling or standardized (z-score transformation) to ensure comparability across features. Categorical variables, where applicable, were encoded using one-hot encoding. To address potential class imbalance, no artificial balancing was initially applied in order to evaluate the intrinsic predictive capacity of the models; however, the imbalance was considered during interpretation of results. Correlations among continuous environmental variables were assessed prior to statistical analyses in order to identify potential multicollinearity among predictors. Since multiple isolates were obtained from individual sampling events, isolates originating from the same sample shared identical environmental measurements. To account for spatial and temporal variability, sampling site and season were included as categorical variables in the analyses.

#### 4.4.2. Model Development

Model performance in predicting bacterial distribution was evaluated using multiple classification algorithms, including artificial neural networks, random forest, support vector machines, extreme gradient boosting, multivariate adaptive regression splines, decision trees, and naïve Bayes [46]. Seven machine learning algorithms were employed for classification: Artificial Neural Network (ANN), Random Forest (RF), Support Vector Machine (SVM), Extreme Gradient Boosting (XGB), Multivariate Adaptive Regression Splines (MARS), Decision Tree (TREE), and Naïve Bayes (NB). The ANN model consisted of a feedforward architecture with a single hidden layer and hyperbolic tangent activation function, while a linear activation function was used in the output layer. RF and XGB models were implemented using ensemble learning techniques based on decision trees, with hyperparameters optimized through grid search. The SVM model employed a radial basis function (RBF) kernel. The MARS model was constructed using adaptive piecewise linear regressions, while the TREE model was based on recursive partitioning. The NB classifier assumed conditional independence among features.

#### 4.4.3. Model Training and Validation

Statistical and machine learning analyses were performed in R. Sampling site and season/time were treated as categorical predictors, while environmental parameters (air temperature, relative air humidity, atmospheric pressure, water temperature, turbidity, pH, electroconductivity, HPK, and BPK) were included as continuous predictors. Sampling site and season were included as categorical variables to account for spatial and temporal variability in the dataset. The dataset was divided into training and testing subsets using stratified sampling to preserve class distribution, typically in a 70:30 ratio. Model performance was evaluated using repeated 10-fold cross-validation with three repetitions in order to reduce overfitting and improve accuracy of performance estimates. The grouped/block validation based on sample origin was not implemented; therefore, isolates originating from the same sample may potentially have been represented in different subsets. Although stratified train–test splitting and repeated cross-validation were applied, the possibility of residual information leakage cannot be completely excluded; therefore, the modeling results should be interpreted as exploratory. Several machine learning algorithms were tested, including artificial neural networks (ANNs), random forest (RF), support vector machines (SVMs), extreme gradient boosting (XGB), multivariate adaptive regression splines (MARSs), decision trees (TREE), and naïve Bayes (NB).

Model evaluation was based on receiver operating characteristic (ROC) optimization and additional performance metrics including accuracy, coefficient of determination (R^2^), root mean square error (RMSE), sum of squared errors (SSE), and average absolute relative deviation (AARD). Continuous predictors used in ANN models were centered and scaled before training. All models were implemented in a consistent computational environment using appropriate machine learning libraries.

#### 4.4.4. Performance Evaluation

Model performance was evaluated using multiple complementary metrics. Discriminative ability was assessed using the area under the receiver operating characteristic curve (AUC), along with corresponding 95% confidence intervals. Classification accuracy, sensitivity (recall), specificity, and precision were calculated to evaluate classification performance. To assess predictive consistency, regression-based metrics including coefficient of determination (R^2^), root mean square error (RMSE), sum of squared errors (SSE), and average absolute relative deviation (AARD) were computed. Performance was assessed using complementary metrics such as AUC, classification accuracy, and error measures (RMSE, SSE, AARD, and R^2^), enabling evaluation of both discriminative power and probabilistic prediction quality [47]. Model accuracy and uncertainty were considered through confidence intervals of AUC values, allowing reliable comparison of algorithm performance under varying environmental conditions [48].

Model calibration was evaluated using the Brier score, where lower values indicate better agreement between predicted probabilities and observed outcomes. Additionally, prediction confidence levels were analyzed by categorizing model outputs into predefined probability intervals (very low to very high confidence).

Receiver Operating Characteristic (ROC) analysis was conducted to evaluate the classification performance of all machine learning models. For each microbial target, predicted probabilities were compared against observed presence/absence data across a range of threshold values to generate ROC curves, plotting the true positive rate (sensitivity) against the false positive rate (1–specificity). The area under the ROC curve (AUC) was calculated as a threshold-independent metric of model discrimination ability. ROC curves and AUC values were derived using cross-validated predictions to ensure strong predictive performance assessment and to minimize overfitting effects.

To identify environmental variables most strongly associated with microbial occurrence, predictor importance was evaluated for RF and XGB models using built-in importance measures based on impurity reduction and gain, respectively. For SVM and ANN models, predictor influence was assessed using sensitivity analysis by examining changes in model outputs following perturbation of input variables. The highest-ranked predictors for each microbial species and model were subsequently extracted and summarized to enable comparative assessment of physicochemical, environmental, spatial, and temporal variables associated with microbial occurrence across models. These analyses were intended to evaluate relative predictor contribution to model performance and do not imply causal relationships. Several potentially relevant environmental factors, including antibiotic concentrations, heavy metals, antibiotic resistance genes, river flow dynamics, and sediment characteristics, were not included in the present study and should be considered in future investigations.

### 4.5. Statistical Analysis

Data analysis included descriptive summaries of bacterial occurrence by sampling site, sample type, and sampling campaign. Machine learning classification models were developed to evaluate associations between environmental predictors and the occurrence of selected bacterial taxa. Model performance was assessed using area under the receiver operating characteristic curve (AUC), accuracy, sensitivity, specificity, precision, F1-score, and probability-based calibration/error metrics, including RMSE, SSE, AARD, and Brier score. Because the dataset was characterized by class imbalance, model outputs were interpreted using multiple complementary metrics rather than accuracy alone. Full model-performance results are provided in the Supplementary Materials. Differences in model performance were interpreted based on combined evaluation of discrimination, calibration, and class balance metrics, rather than relying on a single indicator. Particular attention was given to the trade-off between sensitivity and specificity, especially in the presence of class imbalance. All analyses were conducted using appropriate statistical and machine learning software r, ver. 4.4.3, employing relevant libraries for model development, validation, and performance evaluation.

## 5. Conclusions

This study provides a culture-based assessment of bacterial taxa recovered on selective media from wastewater and surface-water samples collected in the urban section of the Danube River near Novi Sad. The results indicate that wastewater-associated water-quality gradients, including organic pollution indicators, turbidity, pH, and temperature, were associated with the occurrence of several bacterial taxa, particularly *K. pneumoniae*, *A. veronii*, and selected *Pseudomonas* spp. Machine learning models, especially RF and XGB, showed potential for identifying associations between environmental parameters and bacterial occurrence; however, model performance was taxa-dependent and affected by class imbalance and the limited number of sampling campaigns. Because resistance phenotypes and resistance genes were not confirmed, the findings should be interpreted as exploratory and hypothesis-generating rather than as definitive evidence of MDR distribution. Future studies should include standardized antimicrobial susceptibility testing, ESBL/carbapenemase confirmation, qPCR or sequencing-based ARG detection, and broader spatial and seasonal sampling.

## Data Availability

The original contributions presented in this study are included in the article/Supplementary Materials. Further inquiries can be directed to the corresponding author.

## Supplementary Materials

The following supporting information can be downloaded at: https://www.mdpi.com/article/10.3390/antibiotics15060553/s1, Supplementary Materials S1: Table S1. Experimental results; Supplementary Materials S2: Tables S1–S51. Comparative performance, classification outcomes, and prediction confidence of machine learning models across multiple microbial species using site-specific, temporal, and physicochemical predictors; Supplementary Materials S3: Table S1. Main environmental factors influencing microbial occurrence identified by RF, SVM, ANN, and XGB models across all target species.
